# Five new synonyms in *Impatiens* (Ericales, Balsaminaceae) from China

**DOI:** 10.3897/phytokeys.272.177434

**Published:** 2026-04-08

**Authors:** Fang-Ying Wang, Nian Cen, Xin-Xiang Bai, Qin-Qin Yong, Mei-Jun Li

**Affiliations:** 1 College of Forestry, Guizhou University, Guiyang 550025, China Guizhou University Guiyang China

**Keywords:** *Impatiens* L., lectotype, synonymization, taxonomy

## Abstract

Species of *Impatiens* L. on the Yunnan–Guizhou Plateau show exceptionally high diversity, but inadequate early regional surveys have led to persistent taxonomic ambiguity. Critical study of the type and additional specimens, supported by field observations and comparative morphology, demonstrates that: (1) *Impatiens lepida* Hook.f. is conspecific with *Impatiens piufanensis* Hook.f.; (2) *Impatiens sigmoidea* Hook.f. and *Impatiens martinii* Hook.f. represent a single species; (3) *Impatiens abbatis* Hook.f. and *Impatiens leveillei* Hook.f. are not distinct from *Impatiens arguta* Hook.f. & Thomson; (4) *Impatiens foetens* Hook.f. ex H. Lév. and *Impatiens cyanantha* Hook.f. are considered conspecific. In accordance with the International Code of Nomenclature for algae, fungi, and plants (Madrid Code 2025), the above names are reduced to synonymy, and lectotypes are here designated for *I. martinii* and additional species to ensure nomenclatural stability.

## Introduction

The genus *Impatiens* L., comprising more than 1,200 recorded species worldwide (POWO 2026), shows a highly aggregated geographical distribution, predominantly in the tropical and subtropical regions of the Eastern Hemisphere (Grey-Wilson 1980). China constitutes an important distribution center for this genus, with a pronounced regional aggregation that is particularly evident in the hotspot region of Southwest China, notably including South Tibet, the Hengduan Mountains, and the Yunnan–Guizhou Plateau. Recognized as a distinct ecoregion and a critical biodiversity hotspot in Southwest China, the Yunnan–Guizhou Plateau (Qin et al. 2023) exhibits a floristic composition with distinct characteristics, including antiquity, transitional properties, and endemism. The foundational work for later studies was established in the late 19^th^ and early 20^th^ centuries by Western botanists like Joseph Dalton Hooker, who conducted extensive documentation and description of *Impatiens* species in Southwest China (Hooker 1907, 1908a, 1908b, 1910). The inaccessibility of the terrain, compounded by underdeveloped transportation networks, has resulted in significant survey gaps across the region (Chen 1978). Furthermore, constrained by inadequate research conditions, some species have been described exclusively based on single specimens, without systematic population-level surveys. Additionally, the floral structures of *Impatiens* are inherently fragile and highly susceptible to damage during specimen collection and pressing, which substantially elevates the difficulty of observing and identifying key taxonomic characters (Ruchisansakun et al. 2015; Rahelivololona et al. 2018). The aforementioned factors have collectively contributed to substantial taxonomic ambiguities within the genus, encompassing unclear delimitations of certain taxa, synonymy, and taxonomy issues, which further exacerbates the complexity of its taxonomic framework. Based on the latest survey research and the International Code of Nomenclature for Algae, Fungi, and Plants (Madrid Code 2025) (Turland et al. 2025), five new synonyms are hereby proposed, and the lectotypes of the related species are designated.

## Material and methods

Extensive field surveys of *Impatiens* species across the Yunnan–Guizhou Plateau in southwestern China were undertaken. These involved documenting different populations through color photography and recording their morphological characteristics across various phenological stages. Relevant protologues were critically reviewed, verifying key taxonomic information, including type specimen details and geographical distributions. Furthermore, specimens and high-resolution images from herbaria (E, K, P, PE, KUN, GZAC) were examined to compare collection data, distribution records, and morphological traits.

## Taxonomic treatment

### 1. Impatiens arguta

Taxon classificationPlantaeEricalesBalsaminaceae

Hook.f. & Thomson, in J. Proc. Linn. Soc. Bot. 4(no. 15): 137. 1859

14AB023F-2ECA-561C-816F-748CA1AA36C0

Figs 1, 2, 3

= Impatiens leveillei Hook.f. in *Hooker’s Icon. Pl*. 29: t. 2865. 1908. syn. nov. Type: China. Kweichau: Yang Tien, *E. M. Bodinier 493* (holotype: E!, E00313614).
= Impatiens abbatis Hook.f. in *Hooker’s Icon. Pl*. 29: t. 2861 (1908). syn. nov. Type: China. Yunnan au pied du Tsang-chan, S/ Ta-li, *Abbé Delavay 3634* (lectotype designated here: P!, P04542427; isolectotype designated here: P!, P05017683, P05249238).

#### Type.

India • Sikkim, Darjeeling, alt 5–7,000 ped, *J. D. Hooker 101* (lectotype: K!, K000694618; isolectotype: K!, K000694619).

#### Distribution and habitat.

*Impatiens arguta* is distributed in South and Southeast Asia, including India, Myanmar, Bhutan, and Nepal. In China, it occurs in Guizhou Province, where it has been recorded from Guanling County, Xingren City, Zhenfeng County, Panzhou City, and Shuicheng District and Liuzhi Special District of Liupanshui. It typically inhabits valley shrublands and grasslands, moist forest understories, and shaded hillside gullies.

#### Phenology.

Flowering and fruiting occur from July to November.

#### Notes.

First described in 1859 with a type locality in Sikkim (modern-day West Bengal, India), (Hooker 1859) *Impatiens arguta* Hook.f. & Thomson demonstrates significant variation in plant stature, leaf morphology, and floral traits. A continuous color gradient exists among populations, encompassing pure white, pink, pale purple, and purple; further variation is seen in pedicel curvature and spur morphology. The dorsal petal is highly polymorphic. Morphologically, it ranges from broadly ovate to elliptic or orbicular, featuring a mucronate, cuspidate, or emarginate apex and the absence of a horn-like appendage (Abrahamczyk and Steudel 2023). Records from the China Virtual Herbarium (CVH) indicate that most specimens attributed to this species are collected in Southwest China. Taxonomic revisions by Indian scholars have identified several synonyms (Rajeev et al. 2021) and confirmed its widespread occurrence in northeastern India, adjacent to Southwest China, indicating an extensive distribution range for the species.

**Figure 1.**
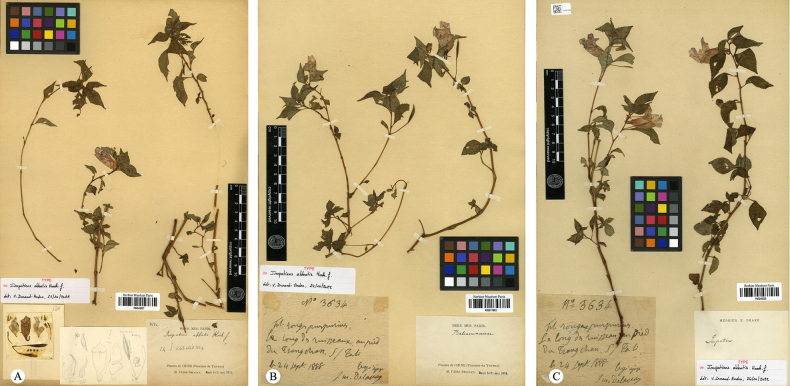
Type specimens of *Impatiens abbatis* Hook.f. **A**. Lectotype of *I. abbatis J. M. Delavay 3634* (P04542427); **B**. Isolectotype of *I. abbatis J. M. Delavay 3634* (P05017683); **C**. Isolectotype of *I. abbatis J. M. Delavay 3634* (P05249238).

In the protologue, *Impatiens abbatis* Hook.f. was noted to resemble *I. arguta* Hook.f. (Hooker 1908a), from which it was distinguished by its much-branched stem, smaller leaves, and the lateral united petals’ distal lobes dolabriform. However, floral dissection of its type material reveals that its morphology, particularly that of the wing petals, is remarkably similar to that of *I. arguta*. Field investigations have corroborated this finding, indicating that stem branching and leaf size are highly susceptible to environmental influences. At the time of its publication, *I. abbatis* was based on three specimens, all bearing the collection number J. M. Delavay 3634, extant at P (P04542447, P05017683, and P05249238), with no holotype designated. Delavay was stationed in the northwest Yunnan region, particularly in the Dali area, where he conducted systematic plant-collecting activities. Between 1883 and 1890, he frequently traveled between Dapingzi, Mosuo Camp (Mo-so-yn), and Dali Fu (Ta-li, Ta-li-fou), with his collecting activities focusing on the foothills of Cangshan Mountain, its upper forest edge, rock faces, and valleys, which became key areas for his botanical surveys (Li 2025). By analyzing the collecting records of Delavay from late September 1888 in Yunnan in the JSTOR specimen database (JSTOR Global Plants 1888), we can reconstruct his itinerary. Specifically, on September 24, 1888, Delavay collected *Hymenophyllum delavayi* at “au pied du Tsang-chan, S/ Ta-li” (the foothills of Cangshan Mountain above Dali). On the following day, September 25, he continued to collect various plants, such as *Sedum beauverdii*, at “au pied du Tsang-chan, au dessus de Ta-li” and sur les rochers au pied du Tsang-chan (rock faces at the base of Cangshan). Delavay’s collecting activities later expanded to the limestone hills near Dali and around Ta Pingzi (Ta-chao / Ta-pin-tze). Given that all known type specimens of *I. abbatis* originate from this collecting period and region, and in the absence of any conflicting locality data, the type locality of the species can be confidently assigned to the Cangshan Mountain area above Dali, Yunnan, China. Herein, specimen P04542447, featuring detailed floral dissections and line drawings consistent with the protologue description, is designated as the lectotype. The remaining specimens, P05017683 and P05249238, are accordingly designated as isolectotypes.

*Impatiens leveillei* Hook.f. was described based on the specimen *E. M. Bodinier 493* collected in Guizhou (Hooker 1908a; Léveillé 1914–1915). According to the protologue and type specimen, the type locality was transcribed as “Yang Tien,” without clear correspondence to modern administrative units. Through analysis of the collector’s itinerary and herbarium records, three specimens directly relevant to the typification of *I. leveillei* were identified. Among them, two sheets of *E. M. Bodinier 492* E (E00010534 and E00313677) labels show a collection date of 16 July and locality “Yang Tien,” consistent with the type locality of *I. leveillei*. These are therefore confirmed as syntopic specimens of this species. Another specimen, *E. M. Bodinier 494* E (E00010625), was collected on 17 July at a locality noted as “Mou-you-se environs.” Based on textual research of modern place names in Guizhou Province, this locality corresponds to the area around Nanhuajiang Town, Guanling Buyi and Miao Autonomous County, Guizhou Province, today. The three aforementioned specimens and the type specimen of *I. leveillei* were collected with only a one-day interval. Considering the extremely underdeveloped transportation in the mountainous areas of Guizhou in the late 19^th^ century, where the transportation of people and materials relied mainly on human porters and horse-drawn loads, and plant collectors typically had a limited daily range of movement, it can be inferred that the collection locality “Yang Tien” should be situated near Huajiang Town, Guanling County, today.

Subsequent verification identified a modern locality named “Yangtian Village” in Huajiang Town, Guanling County. The pronounced phonetic similarity between “Yangtian” and “Yang Tien” provides strong evidence for their transliteration equivalence. Based on this evidence, the type locality “Yang Tien” for *I. leveillei* is definitively established as being located within the area of modern Guanling Buyi and Miao Autonomous County, Anshun City, Guizhou Province.

**Figure 2.**
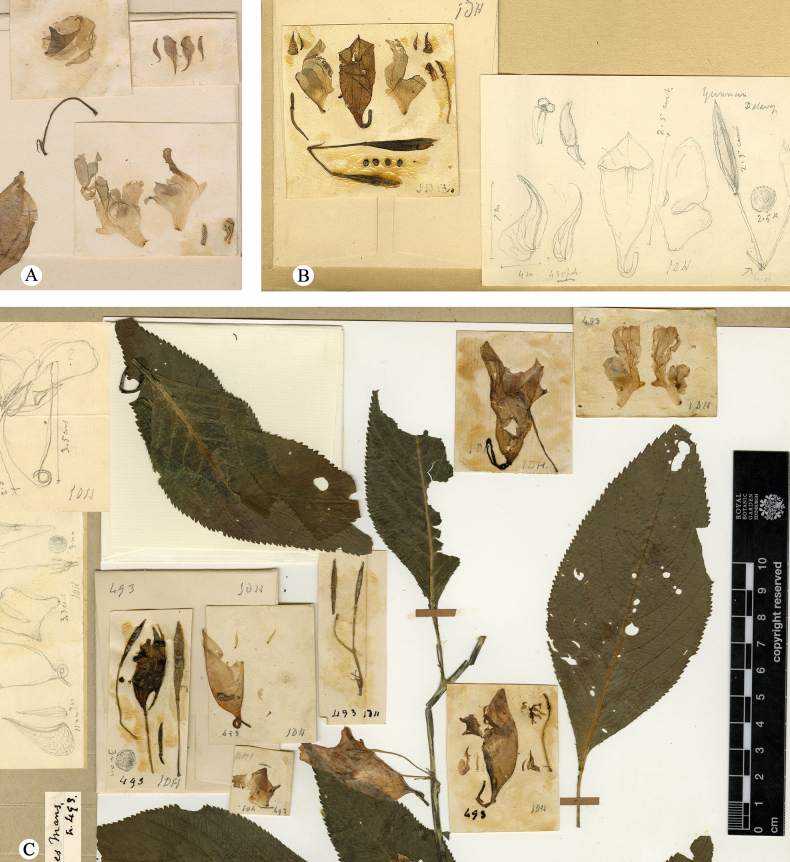
Anatomical diagrams of flower parts in three species type specimens. **A**. Lectotype of *I. arguta J. D. Hooker 101* (K000694618); **B**. Lectotype of *I. abbatis J. M. Delavay 3634* (P04542427); **C**. Holotype of *I. leveillei E. M. Bodinier 493* (E00313614).

According to the protologue and the *Flora of China* (Chen 2001), this species resembles *I. arguta* but is distinguished by its larger, membranous leaves with small crenate margins, along with larger flowers and capsules. However, these size-related traits are unreliable for species delimitation in *Impatiens*, as they are highly plastic in response to environmental conditions. In contrast, *I. arguta*, *I. abbatis*, and *I. leveillei* possess a combination of stable and diagnostically valuable characters, including leaf margin type, a pair of basal leaf glands, a thickened midvein on the abaxial surface of the dorsal petal, a reflexed auricle on the lateral united petals, and setose bracts (Fig. 2A–C). Geographically, the three taxa form a continuous distribution without evident isolation, further supporting their treatment as a single taxonomic entity.

**Figure 3.**
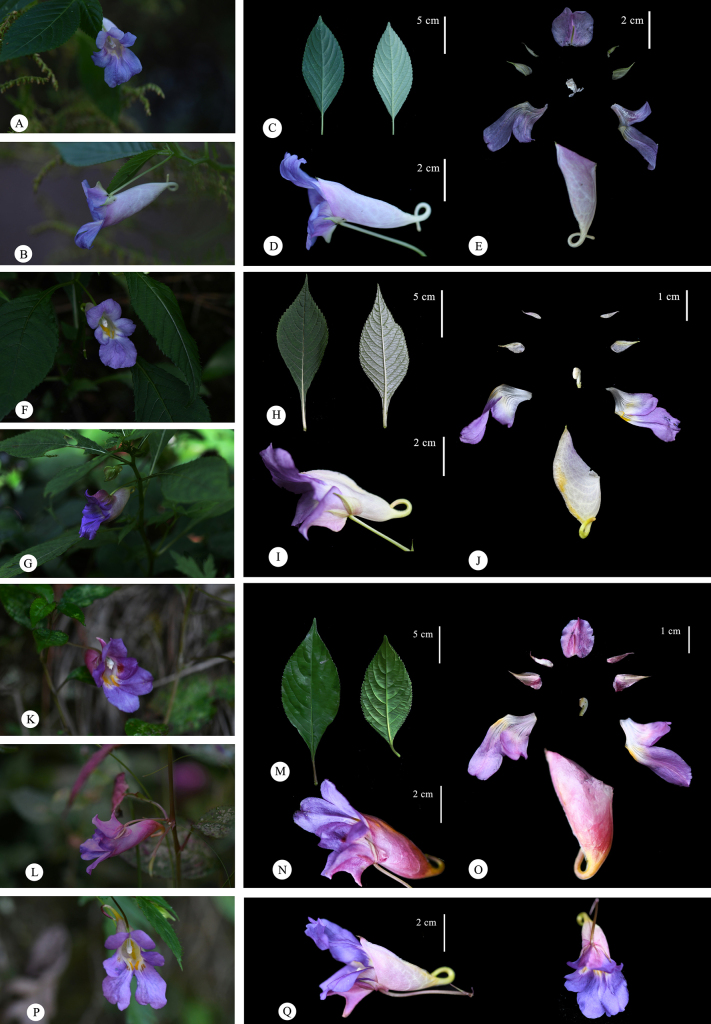
Morphological diversity of different populations of *I. arguta*. Population in Guizhou. **A**. flower in face view; **B**. Flower in lateral view; **C**. Leaf; **D**. Flower; **E**. flower dissected. **F–J**. Population in Kunming City, Yunnan; **F**. Flower in face view; **G**. flower in lateral view; **H**. Leaf; **I**. Flower; **J**. Flower dissected. **K–O**. Population in Dali City, Yunnan (type locality of *Impatiens abbatis*); **K**. Flower in face view; **L**. Flower in lateral view; **M**. Leaf; **N**. Flower; **O**. Flower dissected. **P, Q**. Population in Nyingchi, Tibet Autonomous Region; **P**. Flower in face view; **Q**. Flower in lateral view.

#### Additional specimens examined.

**China, Guizhou**: • Congjiang County, *Li Yongkang 9334* (HGAS); • Libo County, *Luo Yibo 516* (PE). **Yunnan**: • Yangbi County, *Yin Zhijian et al. 0753* (PE); • Shilin County, *Shui Yumin et al. 64189, 64083, 64352* (PE); • Heqing County, *Qin Renchang 23523* (PE); *Luo Yi-bo 44* (PE); • Gongshan County, *Xiaohua Jin & Ting Zhang 098* (PE); *Luo Yi-bo 475* (PE); *Li Hong 533324180418001LY* (SABG); *He Xu et al. 533324180509148LY* (SABG); Dali City, *Sino-Soviet Joint Yunnan Expedition 19* (KUN); *Wang Qiwu 63450 (PE)*; *H.T. Tsai 53922* (IBSC); Shangri-La City, *D.E. Boufford et al. 35270* (PE); *Qinghai-Tibet Expedition 443* (PE); *Institute of Botany, Beijing (Hengduan Mountains Expedition) 02272* (PE); Lushui City, *Institute of Botany, Beijing (Hengduan Mountains Expedition) 499* (PE); *Dulongjiang Expedition 5* (KUN); *Wu Sugong 8098* (KUN); *Guo Shiwei et al. KIBDZL214B03* (KUN); Lijiang City, *Fang Zhendong et al. LDHF-163* (SABG); *Qinghai-Tibet Expedition 189* (KUN); • Yongde County, *Liu Ende 589* (KUN); *Zi Shisheng 18* (KUN); • Yunlong County, *Spice Plant Expedition 101* (KUN); • Yingjiang County, *Zou Jiao et al. 20201029-1* (KUN). **Sichuan**: Panzhihua City, *Gao Xinfen et al. 4038* (PE); *Panzhihua Cycad Expedition 565* (PE); • Muli County, *Qinghai-Tibet Expedition 14875* (PE). **India, Sikkim**: • *J. D. Hooker 101* (NY); *J. D. Hooker 111* (US).

### 2. Impatiens sigmoidea

Taxon classificationPlantaeEricalesBalsaminaceae

Hook.f. in Nouv. Arch. Mus. Hist. Nat. sér. 4, 10: 267.1908

93900098-0F44-5FB1-954F-D4AD4A6DB162

Fig. 4

= Impatiens martinii Hook.f. in *Hooker’s Icon. Pl*. 29: t. 2870. 1908. syn. nov. Type: China. Guizhou: Pin-fa, 25 May 1902, *Cavalerie, P. J. 1329* (lectotype designated here: E!, E00313609); October 1905, *Cavalerie, P. J. 1905* (syntype: E!, E00313610).

#### Type.

China. Guizhou: • Guiyang City, Kao-po, 11 September 1899, *E. M. Bodinier &J. Laborde 2688* (lectotype: E!, E00313668; isolectotype: P!,P00780758). Remaining syntype: China. Guizhou: • Pin-fa, 21 August 1902, *Cavalerie, P. J. 287* (E00313667).

#### Distribution and habitat.

*Impatiens sigmoidea* occurs in Guizhou Province, where it has been recorded from Guiyang City, Longli County, and Qiannan Buyi and Miao Autonomous Prefecture. It typically grows on gentle slopes along streams or in evergreen broad-leaved forests.

#### Phenology.

Flowering and fruiting occur from May to October.

#### Notes.

**Figure 4.**
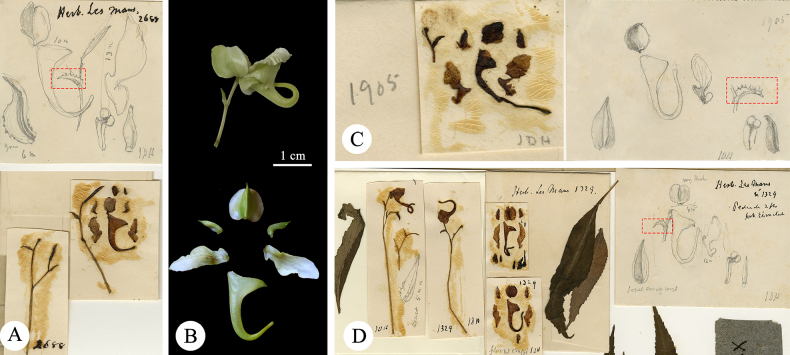
Comparative study of floral structures between *I. sigmoidea* Hook.f. and *I. martinii* Hook.f. **A**. Lectotype of *I. sigmoidea Bodinier, E. M. & Laborde, J. 2688* (E00313668); **B**. Floral dissection of *I. sigmoidea*; **C**. Syntype of *I. martinii Cavalerie, P. J. 1905* (E00313610); **D**. Lectotype of *I. martinii Cavalerie, P. J. 1329* (E00313609) (hand-drawn illustration of a bud with teeth marked in red box).

*Impatiens martinii* Hook.f. was described by J. D. Hooker in September 1908 (Hooker 1908a), based on specimens collected by Cavalerie in Pin-fa. Its Chinese name derives from the toothed margins of its bracts. The protologue cited two syntypes (Fig. 4C–D) that exhibit floral color variation ranging from yellow to white, which is attributable to natural interpopulation chromatic transitions. As no holotype was designated in the protologue, the specimen *Cavalerie, P. J. 1329* E (E00313609), which fully matches the morphological description in the protologue and includes detailed floral dissections and line drawings, is here designated as the lectotype. The other specimen, *Cavalerie, P. J. 1905* E (E00313610), is accordingly designated as an syntype.

*Impatiens sigmoidea* Hook.f. was formally described in April 1908 based on two type specimens collected from Gao-po and Pin-fa, Guizhou Province (Hooker 1908b). No subsequent records were documented until it was rediscovered at the type locality in 2018 (Huang et al. 2023a). Observations of wild-living plants of this species reveal that the middle and upper parts of the stem are covered with white to pink hairs; bracts are borne at the base, middle, or upper-middle part of the pedicel and are either persistent or easily deciduous. It is also noted that the bract margins, like those of *I. martinii*, are toothed. Compared with other species in the genus, *I. sigmoidea* possesses a suite of distinctive morphological characters, including a white midvein of the leaf, conspicuously serrate bract margins, lateral sepals with a prominent “S”-shaped curve, a spiral shell-like structure of the dorsal petal, and a spiral shell-like or cucullate projection on the abaxial surface of the midrib. Comparison of the type specimens, floral dissections, and line drawings (Fig. 4A–D) reveals the following morphological distinctions between the protologue descriptions of *I. sigmoidea* and *I. martinii*: bracts entire (vs. *I. martinii* has serrate), flowers greenish white (vs. *I. martinii* has white or yellow), lateral united petals bilobed (vs. *I. martinii* has trilobed), dorsal petal rounded (vs. *I. martinii* has cucullate), and midrib abaxially bearing a broad cristate projection (vs. *I. martinii* has a beaked keel). However, the floral morphology, particularly the dissected structures, exhibits remarkable similarity between the two taxa, including the “S”-shaped curved lateral sepals, toothed bract margins, and spiral shell-like dorsal petal. Given the absence of consistent diagnostic characters and the overlapping type localities, combined with extensive field observations, it is proposed that *I. sigmoidea* and *I. martinii* represent a single species.

#### Additional specimens examined.

**China, Guizhou**: • Huaxi District, *Bai Xinxiang GP2018051501* (GZAC); Longli County, *Bai Xinxiang HZG2018051901* (GZAC!).

### 3. Impatiens piufanensis

Taxon classificationPlantaeEricalesBalsaminaceae

Hook.f. in Hooker’s lcon. PI.29:t.2869.1908

778BB8A3-EF30-5150-BAC9-D9A5FD7F4262

Figs 5, 6

= Impatiens lepida Hook.f. in *Hooker’s lcon. PI*.29: t.2867. 1908. syn. nov. Type: CHINA. Guizhou: Piu-fa (=pingfa), 27 October 1903, *Cavalerie, P. J. 1441* (lectotype designated here: E!, E00313616, the lower sepal funnelform specimen in the admixture).

#### Type.

China. Guizhou: • Piu-fa (=pingfa), *Cavalerie, P. J. 314* (holotype: E!, E00313603; isotype: K!, K000694588).

#### Distribution and habitat.

*Impatiens piufanensis* occurs in Sichuan, Yunnan, Guangxi, Hubei, Hunan, Chongqing, and Guizhou provinces, where it has been recorded from Duyun City, Fuquan City, Anlong County, Shibing County, Kaiyang County, Huangping County, Chishui City, Suiyang County, and Jinsha County. This species typically grows in forests, damp areas along streams, and rocky crevices.

#### Phenology.

Flowering and fruiting period: July to November.

#### Notes.

**Figure 5.**
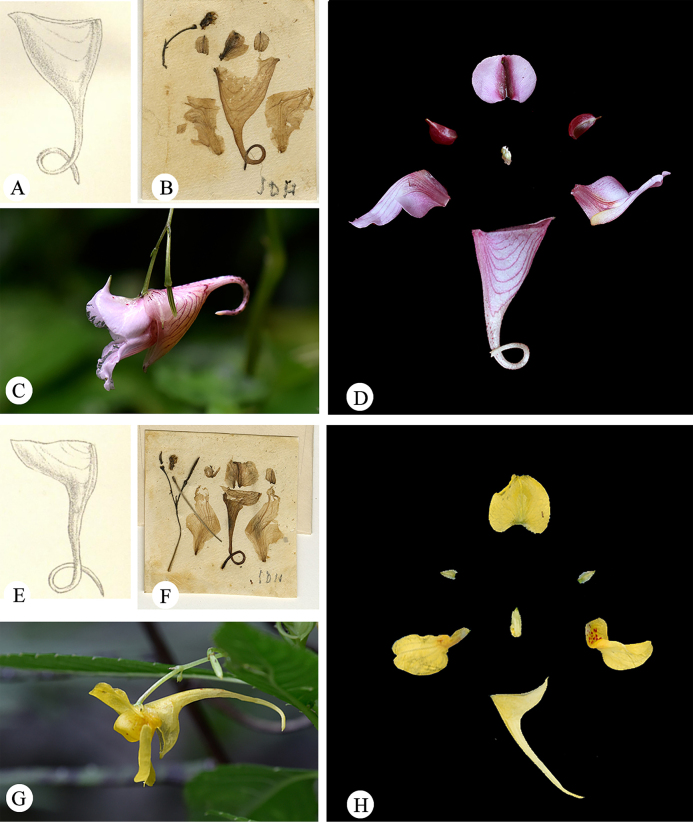
Comparison of flower morphology of *Impatiens lepida*, *I. piufanensis* and *I. procumbens*. **A**. *I. lepida*, the lower sepal funnelform specimen of the protologue; **B**. The lower sepal funnelform specimen of *I. lepida* (E00313616); **C**. *I. piufanensis*, flower in lateral view; **D**. *I. piufanensis*, flower dissected; **E**. *I. lepida*, the lower sepal cymbiform specimen of the protologue; **F**. The lower sepal cymbiform specimen of *I. lepida* (E00313616); **G**. *I. procumbens*, flower in lateral view; **H**. *I. procumbens*, flower dissected.

**Figure 6.**
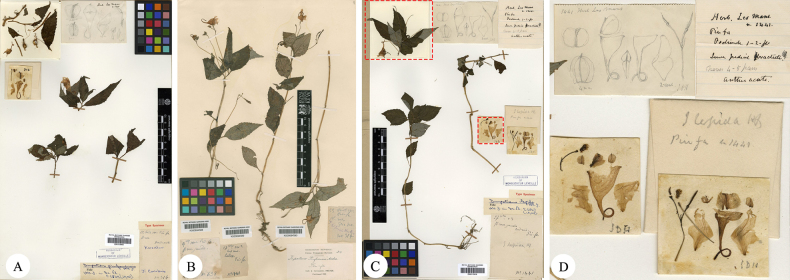
Comparison of type specimens between *Impatiens piufanensis* Hook.f. and *Impatiens lepida* Hook.f. **A**. Holotype of *I. piufanensis Cavalerie, P. J. 314* (E00313603); **B**. Isotype of *I. piufanensis Cavalerie, P. J. 698* (K000694588), *Cavalerie, P. J. 1441* (K000694589), *Cavalerie, P. J. 314* (K000694590); **C, D**. Type specimen of *I. lepida Cavalerie, P. J. 1441* (E00313616) and its floral morphology (red box indicates the funnel-shaped labellum specimen).

*Impatiens lepida* Hook.f. was described by J. D. Hooker based on specimens collected by Cavalerie and others from Pin-fa, Guizhou (Hooker 1908a). Examination of the type material revealed that the specimen label records the flower color as “rose-jaunâtre.” The floral morphological description and dissections indicate the presence of two flowering individuals: one with a single flower and another with two flowers on the pedicel, also showing distinctly different lower sepal shapes. Within *Impatiens* L., the lower sepal is considered a stable character for species delimitation, and no transitional forms between two distinct shapes have been documented. Hooker himself expressed doubt in the protologue regarding the occurrence of two flower colors and lower sepal shapes in this species. As shown in the floral dissections from the right middle part of the holotype of *I. lepida*, *Cavalerie, P. J. 1441* (E00313616) housed at E (Fig. 6D), one lower sepal is funnel-shaped, while the other is boat-shaped. Extensive field surveys in the type locality of *I. lepida* have failed to identify any population exhibiting both funnel-shaped and boat-shaped sepal shapes. Based on the above evidence, it is concluded that this specimen comprises two distinct taxa. Regarding the admixture in the type specimens of *I. lepida*, extensive field surveys conducted in the Pin-fa area confirmed that floral anatomical specimens exhibiting a navicular lower sepal are attributable to *Impatiens procumbens* Franch., a species widely distributed in this region (Hooker 1908b; Huang et al. 2023b) (Fig. 5A–C). Field surveys further revealed a high degree of morphological similarity between *Impatiens piufanensis* Hook.f. and *I. lepida*, which share Pin-fa as a type locality. Examination of the *I. piufanensis* specimen K000694588 (K) revealed that it integrates three accessions (K000694588, K000694589, K000694590), all collected by Cavalerie under numbers 698, 1441, and 314. The label for No. 698 records flower color as “yellow or red,” and all three plants exhibit funnelform labella. In contrast, field observations document continuous variation in lower sepal shape within populations of this taxon, ranging from funnelform to saccate. Comparative morphological analysis detected no consistent differences between *I. lepida* and *I. piufanensis* (Fig. 6A–D). In view of their overlapping type localities and morphological continuity, *I. lepida* and *I. piufanensis* are accordingly treated as a single species.

In accordance with Articles 9.3 and 9.14 of the ICN, no holotypes were designated for either species upon their original publication. Herein, the specimen *Cavalerie, P. J. 314* E (E00313603) is formally designated as the lectotype of *Impatiens piufanensis*, with the duplicate specimen *Cavalerie, P. J. 314* K (K000694588), recognized as an isolectotype. Additionally, the portion of the specimen *Cavalerie, P. J. 1441* E (E00313616) bearing funnelform labella is designated as the lectotype of *I. lepida* (Fig. 6C, red frame).

#### Additional specimens examined.

**China, Guizhou**: • Guiyang City, *Bodinier 2463* (P); • Guanshanhu District, *Bai Xinxiang RFS-000248* (GZAC); • Guiding County, *Bai Xinxiang RFS-00046* (GZAC); • Fuquan City, *Bai Xinxiang RFS-000301* (GZAC); • Huishui County, *Bai Xinxiang RFS-000089* (GZAC); • Leishan County, *Jia Chaopo et al. 50536* (PE); • Rongjiang County, *Liu Zhonghua & Pu Lihua 23143* (BJFC).

### 4. Impatiens cyanantha

Taxon classificationPlantaeEricalesBalsaminaceae

Hook.f. in Hooker’s lcon. PI.29:t.2866.1908

69CFCBFF-0081-5978-9951-B155B76D1BDE

Figs 7, 8, 9

= Impatiens foetens Hook.f. ex H.Lév. in *Fl. Kouy-Tcheou* 176 (1914–15). syn. nov. Type: CHINA. Guizhou: Piu-fa (=pingfa), Nov.1908, *Cavalerie, P. J. 3613* (lectotype designated here: K!, K000694642; isolectotype designated here: K!, K000694643).

#### Type.

China • Kweichau, Sep. 1904, *Esquirol, Joseph Henri (Rev. Père) 226* (holotype: E!, E00313642).

#### Distribution and habitat.

*Impatiens cyanantha* is distributed in the Guangxi Zhuang Autonomous Region, Sichuan, Hunan, Jiangxi, and Guizhou provinces, where it has been recorded from Duyun City, Weining County, Dafang County, Nayong County, Hezhang County, and Panzhou City, as well as Zhongshan District of Liupanshui.

#### Phenology.

Flowering and fruiting period: July to October.

#### Notes.

*Impatiens foetens* Hook.f. ex H. Lév. was originally published in the “Flore du Kouy-Tchéou” (1914–1915), based on specimens collected by Cavalerie from Pin-fa (Léveillé 1914–1915). The protologue cites specimen number *Cavalerie, P. J. 3613*, though no holotype was designated at the time of publication. Investigation of herbarium material identified two original specimens at K (K000694642 and K000694643). Morphological examination and label analysis confirm that K000694642 bears the annotations “fleurs rose-violet” and “odeur forte désagréable,” corresponding to the protologue reference to a “Plante à odeur fétide.” However, extensive long-term field observations of wild *Impatiens* have not recorded any species with a conspicuous odor. This discrepancy warrants further validation through examination of living material in future studies. Examination of this specimen reveals the following morphological characteristics: leaves alternate, elliptic, elliptic with coarsely crenate-serrate margins bearing minute setae between teeth. Inflorescence racemose, with peduncles axillary; bracts ovate-lanceolate. Lateral sepals 2, ovate with acuminate apex; dorsal petal orbicular, bearing a slender midrib (notably, floral dissection shows the dorsal petal midrib only slightly thickened and acute apically, whereas the accompanying line drawing depicts a distinct cristate crest and a beaked apex, suggesting illustrative inaccuracy). Lateral united petals bilobed; upper lobe linear with obtuse apex. Lower sepal funnel-shaped, gradually attenuating into a slender basal spur; anthers obtuse; capsule fusiform; seeds ovoid. This specimen comprises comprehensive floral dissections, detailed ink drawings, and clearly preserved floral morphological characters, thereby providing a robust basis for species delineation. K000694643 is likewise a type specimen, but it lacks such critical diagnostic elements. K000694642 is hereby designated as the lectotype for this taxon, and K000694643 is designated as an isolectotype.

*Impatiens cyanantha* Hook.f. was formally described by J. D. Hooker in 1908, based on the specimen *Esquirol, Joseph Henri (Rev. Père) 226* collected in Guizhou (Hooker 1908a). The specimen was gathered in September 1904, though no precise type locality was provided in the original publication. To clarify the geographical scope of the type locality, the 1904 field itinerary of the collector Joseph Henri Esquirol in Guizhou was reconstructed: in May 1904, Esquirol departed from Guiyang and traveled southwest to the Wangmo area, with subsequent activities extending to the vicinity of Zhenfeng County; in June, his collections were concentrated in Huajiang Town, Wangmo County, and Longli County; by August, his focus had shifted to locations within Zhenfeng County; in September, based on the collection data from extant herbarium specimens, Esquirol’s locality is recorded as “Ko-tchang-keou.”

**Figure 7.**
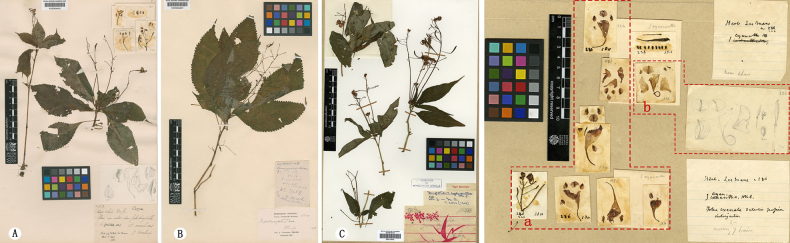
Comparison chart of type specimens between *I. foetens* and *I. cyanantha*; **A**. Lectotype of *I. foetens Cavalerie, P. J. 3613* (K000694642); **B**. Isolectotype of *I. foetens Cavalerie, P. J. 3613* (K000694643); **C**. Holotype of *I. cyanantha Esquirol, Joseph Henri (Rev. Père) 226* (E00313642).

**Figure 8.**
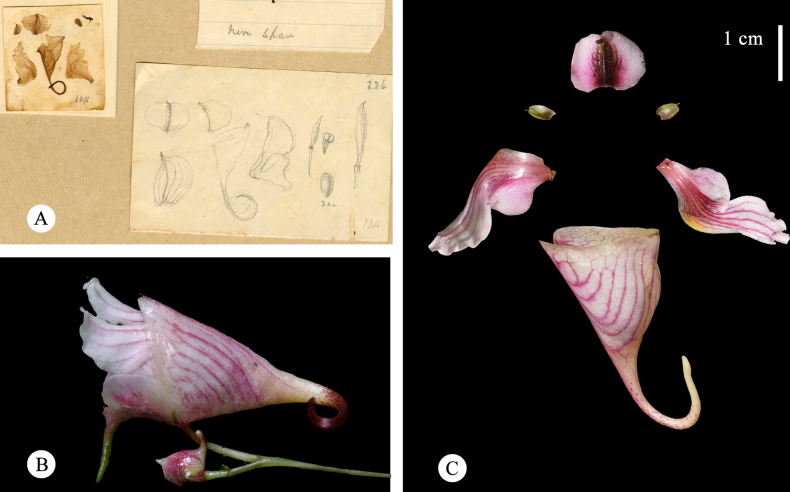
Comparison of flower morphology of *I. cyanantha* and *I. piufanensis*. **A**. *I. cyanantha*, the lower sepal funnelform specimen of *Esquirol, Joseph Henri (Rev. Père) 226* (E00313642); **B**. *I. piufanensis*, flower in lateral view; **C**. *I. piufanensis*, flower dissected.

Upon examination of the floral morphology of the type specimen E00313642, the floral anatomical section reveals clear evidence of taxonomic admixture. Based on the floral structures illustrated in parts a and b (Fig. 7C), the anatomical sample comprises two distinct taxa: (Fig. 7C: a), which corresponds to the widely accepted concept of *I. cyanantha* in current taxonomy, whereas the floral morphology of (Fig. 7C: b) matches the protologue description of wing petals with lateral united petals basal lobes orbicular; distal lobes dolabriform. Subsequent field surveys (Fig. 7C: b) confirm that this portion closely resembles *I. piufanensis*, a species with its type locality in Pin-fa (Fig. 8). Sympatric populations of *I. piufanensis* and *I. cyanantha* have also been documented on Doupeng Mountain, Duyun City.

**Figure 9.**
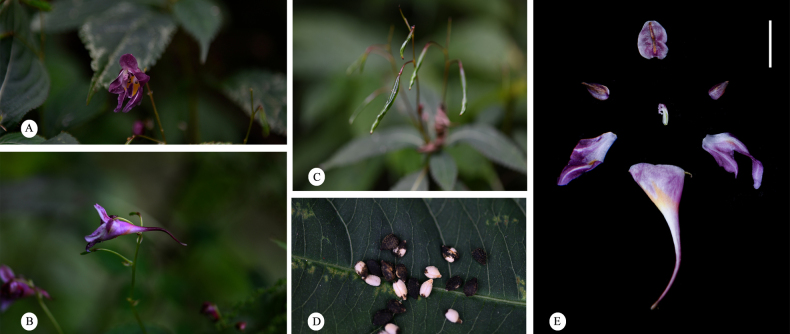
*Impatiens cyanantha*. **A**. Flower in face view; **B**. Flower in lateral view; **C**. Capsule; **D**. Seed; **E**. Flower dissected.

Based on these findings, the morphological description of *I. cyanantha* is hereby emended as follows: the midrib of the dorsal petal is slightly thickened; the spur is straight, slightly curved, or curved; the basal lobes of the lateral united petals are oblong, and the distal lobes are linear. In accordance with Article 9.3 of the ICN (2025), which stipulates that a lectotype may be designated when the original type material comprises multiple taxa, the floral anatomical portion of specimen E00313642 (Fig. 7C: a) is formally designated as the lectotype of *I. cyanantha*.

Examination of type specimens, comparative morphological analyses, and distribution patterns indicate that *I. foetens* and *I. cyanantha* share consistent key taxonomic characters (Table 1). Both species possess racemose inflorescences and exhibit no significant differences in the morphology of the dorsal petal or the lateral united petals, which are critical diagnostic features within *Impatiens*. Other traits, leaf shape, sepal morphology, lower sepal structure, and fruit and seed characteristics, also show high similarity, with minor variations such as leaf size, pedicel length, or bract shape falling within the expected intraspecific range. Distribution data support this close relationship, with *I. foetens* recorded from Pin Fa, Guizhou, and *I. cyanantha* from Wangmo County, Zhenfeng County, and Duyun City, Guizhou. Collectively, these findings demonstrate that the two names refer to a single taxon. In accordance with the principle of nomenclatural priority under the ICN, *I. foetens* is here treated as a synonym of *I. cyanantha*.

**Table 1.** The comparison of discriminative morphological characters between *Impatiens foetens* and *I. cyanantha* (data from protologue and type specimen).

Characteristics	*I. foetens*	*I. cyanantha*
Odor	Reported as strongly fetid in protologue, not confirmed in field	No obvious odor
Leaf	Alternate, elliptic, margin with coarse rounded serrations	Alternate, ovate to ovate-lanceolate, rounded serrations, 5–15 cm long, 2–5 cm wide, petiole 1–3 mm long
Flower	Fleus rose-violet; raceme, axillary; pedicels ca. 1.5 cm, bracts ovate-lanceolate	Blue or purple-blue; raceme, axillary; pedicels ca. 1 cm, slender bracts lanceolate
Lateral sepal	Two, orbicular, 6 mm long	Two, orbicular, inequilateral, leathery, ca. 5 mm long, ca. 4 cm wide
Dorsal petal	Orbicular, abaxial midvein fine, 6 mm in diam	Orbicular, abaxial midvein fine, 6 mm in diam
Lateral united petals	ca. 15 mm long, basal lobes orbicular, distal lobes linear.	ca. 16 mm long, basal lobes orbicular, distal lobes linear.
Lower sepal	Saccate	Saccate
Anther	Obtuse	Obtuse
Fruit	Capsule fusiform, 4 mm long	Capsule narrowly fusiform, 3–5 mm long
Seeds	Ovoid, 4–4.5 mm long	Ovoid, ca. 5 mm long

#### Additional specimens examined.

**China. Guizhou**: • Zhenfeng County, *Y. Tsiang 4360* (IBSC); • Leishan County, *Liu Quanru et al. LGS2003036, LGS2003156, LGS2003157* (BNU); *Qiannan Expedition 3549* (PE); • *Qiannan Expedition Cao Ziyu 3549* (PE); Pu’an County, *Anshun Expedition 1193*, *1196, 1606* (PE); • Nayong County, *Bijie Expedition 676* (PE); • Shibing County, *Wuling Mountain Expedition 1763, 2481* (PE); • Jiangkou County, Sino-American Guizhou *Botanical Expedition 1016* (PE); • Songtao County, *Sino-American Guizhou Botanical Expedition 1887* (PE); • Yinjiang County, *Sino-American Guizhou Botanical Expedition 1442* (PE); *Jian Zhuopo et al. 30922* (PE); • Panzhou City, *Anshun Expedition 1031, 1251* (PE); • Dafang County, *Bijie Expedition 896* (PE); • Congjiang County, *Liu Kaitao et al. 522633190912330LY* (GZTM). **Yunnan**: • Nanjian County, *Chen Yaping et al. EM178, EM184* (KUN); • Luchun County, *Shui Yumin et al. 70216, 70303, 70356* (PE); *Tao Deding 851, 1080* (KUN); • Jingdong County, *Yang Guoping 255* (PE); *Xu Sugui 5507* (PE, KUN); *Sun Hang 72* (KUN); • Chuxiong City, *M.K. Li 0230* (PE); • Yangbi County, *Qin Renchang 25315* (KUN). **Chongqing**: • Xiushan County, *West China Academy of Sciences Sichuan Botany 3938* (PE). **Guangxi**: • Ziyuan County, *Ziyuan County General Survey Team 450329160810012LY, 450329160809017LY* (IBK).

## Discussion

Plant taxonomy, a discipline steeped in historical tradition, counts species delimitation among its fundamental tenets. This scholarly endeavor hinges intrinsically on the systematic observation and rigorous scrutiny of plant morphological traits. Through meticulous dissection and comparative analysis of characters across key plant organs: roots, stems, leaves, flowers, and fruits, taxonomists delineate species boundaries. The comprehensiveness and precision of morphological investigations directly underpin the scientific rigor and robustness of species circumscription. Accurate characterization of morphological attributes thus constitutes the cornerstone of plant taxonomic inquiry (Hong 2025).

Yet *Impatiens* poses distinctive taxonomic challenges. Endowed with remarkable species richness, its members are typified by succulent tissues and exquisitely delicate floral structures. These biological peculiarities confound taxonomic endeavors, substantially elevating the complexity of specimen identification and rendering *Impatiens* one of the most taxonomically intractable lineages (Peng 2019). In this context, herbarium specimens assume paramount significance. They serve not only as the definitive basis for the publication of new species names but also as critical touchstones in subsequent taxonomic revisions and assessments of species validity. By facilitating the verification of morphological characters and the corroboration of nomenclatural applications, specimens furnish indispensable support for upholding consistency and standardization in species delimitation.

## Supplementary Material

XML Treatment for Impatiens arguta

XML Treatment for Impatiens sigmoidea

XML Treatment for Impatiens piufanensis

XML Treatment for Impatiens cyanantha

## Additional information

### Use of AI

No use of AI was reported.

### Author ORCIDs

Fang-Ying Wang https://orcid.org/0009-0000-3791-2474

Nian Cen https://orcid.org/0009-0005-3419-7280

Xin-Xiang Bai https://orcid.org/0000-0003-2449-6664

Mei-Jun Li https://orcid.org/0009-0001-3433-4987

### Data availability

All of the data that support the findings of this study are available in the main text.
